# Development and validation of a scale of cyberbullying and online aggressive conduct in Brazilian adolescents

**DOI:** 10.3389/fpsyt.2026.1759871

**Published:** 2026-05-05

**Authors:** Rosana Fanucci Silva Ramos, Wanderlei Abadio de Oliveira, Claudio Romualdo, Makilim Nunes Baptista, Luciana Bertoldi Nucci, José Eugenio Rodríguez-Fernández, Laura Soares da Silva, Evelin Moreira Freires, Amanda Severo Lins Vitta, Fernando Ferreira Semolini, Adriana Scatena, Denise De Micheli, André Luiz Monezi Andrade

**Affiliations:** 1 School of Life Sciences, Pontifical Catholic University of Campinas, Campinas, Brazil; 2 Faculty of Education Sciences, University of Santiago de Compostela, Santiago de Compostela, Spain; 3 Psychology Section, Santo André Medical Center, Santo André, Brazil; 4 Department of Psychobiology, Universidade Federal de São Paulo, São Paulo, Brazil

**Keywords:** adolescents, cyberbullying, mental health, psychometric validation, risk assessment

## Abstract

**Background:**

Cyberbullying (CYB) is an increasing problem, especially among young people, requiring new ways to measure this construct. Currently, there are gaps in the development of a scale to validate the identification of CYB. Therefore, this study aimed to develop and validate the Scale of Cyberbullying and Online Aggressive Conduct (SCOAC), considering a sample of 642 adolescents (11–17 years old).

**Methods:**

For the internal structure, we conducted Exploratory Factor Analysis (EFA) and Network Analysis (NA) on 60% of the sample, followed by Confirmatory Factor Analysis (CFA) on the remaining 40%, with multigroup invariance analyses by gender. We also evaluated external evidence, including criterion validity (using the Florence Cybervictimization Inventory - FCBVS), convergent validity (SAS-SV, SPAI-SF, STDS, IAT, PIUQ-9), and nomological validity (DASS-21, DERS-18, UPPS-P, aggression, quality of life, CRAFFT/CESARE). Predictive validity was examined with an ROC curve based on risk classes from Latent Profile Analysis (LPA).

**Results:**

EFA indicated KMO = 0.890 and explained 72.4% of the variance; the final 3-factor solution with 17 items (Threat; Defamation/Exposure; Emotional Problems) was confirmed in the CFA (CFI = 0.991; TLI = 0.989; RMSEA≈0.030; SRMR = 0.048). The reliability of the SCOAC was high (total α=0.915; total ω=0.919; subscales α=0.837–0.873). In criterion validity, the total SCOAC score correlated with the FCBVSs instrument (ρ = 0.649). In convergent validity, we found higher correlations with indicators of smartphone use/messaging. In the nomological validity analysis, correlations were moderate for anxiety (ρ=0.437), stress (ρ=0.404), and depression (ρ=0.387). The ROC analysis showed an AUC of 0.85 (95% CI 0.82–0.88) and a cutoff point of ≥8. 29.9% were classified as having signs of CYB and demonstrated greater emotional distress, impulsivity, emotional dysregulation, aggression, poorer quality of life, and increased substance involvement compared to others.

**Conclusions:**

The SCOAC has a stable structure, high reliability, strong evidence of validity, and good screening accuracy for CYB, making it a brief, practical tool for identifying cyber risks among adolescents.

## 1 Introduction

Cyberbullying (CYB) is a form of emotional and social violence where the perpetrator intentionally aims to intimidate, defame, embarrass, or exclude individuals or groups using various online platforms, such as social media, messaging apps, and games (1, 2). Especially in Brazil, data from the Brazilian Institute of Geography and Statistics, gathered from a probabilistic, representative sample of Brazilian teenagers, showed that approximately 13% reported experiencing some form of online discrimination or aggression (3).

These data are critical because increased access and early usage, particularly via mobile devices, can raise exposure to online risks (4). A report by the Brazilian Internet Steering Committee indicated that smartphone use among children and teenagers (ages 9–17) increased from 85% in 2015 to 97% in 2023 (5). According to the same report, approximately 20% of adolescents access the internet exclusively through smartphones, and nearly 40% of those from lower socioeconomic groups do so as well. For these individuals, parents of adolescents from lower economic backgrounds often cannot supervise their children’s online activities in person due to longer work hours, especially when these teens are online outside of school. Another challenge is that lower levels of education and digital literacy among these parents can make it more difficult for them to recognize the potential online risks facing their children (6, 7). Although extensive research links CYB with emotional distress in teens (8, 9), “cyberbullying” is not a formal diagnostic category in the DSM-5 or ICD-11 (10). This is because these manuals categorize mental disorders but do not classify CYB as formal clinical entities, which limits classification based on public policy and screening tools. Regarding public policy, understanding CYB has recently advanced in Brazil with the enactment of Law No. 14,811/2024, which criminalizes bullying and cyberbullying and incorporates prevention measures into the educational system.

Regarding detection tools, despite the growing number of studies on CYB, some authors have noted significant limitations in psychometric assessment. In a systematic review of 50 studies, the authors found notable discrepancies in the terms, definitions, and behaviors used to conceptualize CYB (11). Another systematic review of over 64 scales revealed that only 36% (n = 23) explicitly focused on CYB victimization, and only 15 studies followed the recommended guidelines for scale development (12). Additionally, the authors observed that fewer than 30% of the studies even defined the CYB conceptualization they used, and only 11 included both exploratory and confirmatory factor analyses to evaluate the same instrument.

Taken together, these data highlight the need to develop new CYB screening tools for various purposes. In two systematic reviews assessing the impacts of CYB prevention programs, the authors noted that part of the positive results could have been due not to the quality of the intervention but to the inaccuracy of the instruments used to evaluate CYB, as some of them were not validated or specific to identify this construct (13, 14).

In this context, our study helps fill a scientific gap by developing and assessing the psychometric properties of a specific scale for cyberbullying (Cyberbullying and Online Aggressive Behavior Scale – SCOAC). In addition to its psychometric contribution, the SCOAC was developed as a brief screening tool for adolescents, with potential use in school and clinical settings (for psychologists, educational psychologists, and psychiatrists). By detecting experiences of cyberbullying and their emotional and psychosocial correlates, we hope that the SCOAC will aid in early identification of adolescents at higher risk and also serve as a tool to evaluate the effectiveness of intervention programs in this field.

The aims of our study were (1): to create the SCOAC and gather initial evidence of face and content validity; (2) to analyze its internal structure using exploratory methods (Exploratory Factor Analysis and Network Analysis) and confirmatory methods (Confirmatory Factor Analysis); (3) to test multigroup invariance (by sex) of the confirmatory model; (4) to determine the internal consistency of the SCOAC (using Cronbach’s α and McDonald’s ω coefficients); (5) to collect evidence of criterion, convergent, and nomological validity; (6) to evaluate predictive validity through ROC curves and establish a cutoff point for case screening; and (7) to describe the psychosocial profile of participants based on scale scores.

We hypothesized that: (1) the SCOAC would demonstrate robust face and content validity, effectively representing the CYB construct; (2) its internal structure would be supported by exploratory (EFA and network analysis) and confirmatory models, with invariance between boys and girls; (3) the SCOAC would exhibit high internal consistency (α and ω exceeding 0.80); (4) external validity evidence would show strong correlations with CYB measures (criterion validity) and moderate correlations with related constructs (convergent and nomological validity); (5) ROC analysis would indicate good diagnostic accuracy, identifying a cutoff point for CYB detection; and (6) adolescents classified as CYB would display significantly higher frequencies of online aggressive behaviors, greater emotional vulnerability (anxiety, depression, and stress), increased aggression levels, and a lower quality of life compared to other adolescents.

## 2 Methods

This study was approved by the Ethics Committee of the Pontifical Catholic University of Campinas (No. 6,087,495; CAAE: 67886023.1.0000.5481), and all research was conducted in accordance with the principles of the Declaration of Helsinki and the resolutions of the Brazilian National Health Council, which guide ethical procedures in research involving humans. All participants and their parents or guardians signed an informed consent form.

### 2.1 Design

To develop the SCOAC, we followed several steps, including establishing a theoretical foundation, item development, content analysis, a pilot study, and an examination of psychometric properties. Generally, the process involved: (1) defining the construct conceptually and identifying the target population; (2) creating an initial item bank based on literature and psychoeducational materials about cyberbullying; (3) assessing the content and clarity of items by expert judges; (4) analyzing adolescents’ understanding and practical relevance of the items; and (5) empirically testing the internal structure, reliability, and validity of the refined instrument.

### 2.2 Conceptual framework and item generation

In this step, we conducted an integrative literature review to develop the conceptual framework and create the items. In addition to scientific articles, we examined booklets and psychoeducational materials used in cyberbullying prevention programs in Brazil, which helped develop an initial 30-item version of the instrument. We designed the initial items based on the theoretical definition of the construct, findings from the literature review, and recurring situations described in psychoeducational and prevention materials, rather than directly adapting a pre-existing validated instrument.

Among the theoretical models used to explain cyberbullying behaviors, we chose the Lifestyle-Routine Activities Theory (L-RAT), which combines Routine Activity Theory (15) and Lifestyle-Exposure Theory (16). This framework guided three key aspects of the SCOAC development: the conceptual definition of cyberbullying, the identification of target behaviors to include in the instrument, and the organization of the initial item pool. From this perspective, cyberbullying in adolescence can be seen as a form of online victimization that occurs in digital environments marked by frequent exposure, easier access between victim and perpetrator, limited protective oversight, and the ongoing presence and sharing of harmful content. Therefore, certain online routines may increase the chances of contact between suitable targets and motivated offenders, especially when parental supervision or other protective mechanisms are weak or lacking.

Therefore, in a digital environment, exposure to advertising and fake news, the persistence and replicability of content, and gaps in parental mediation or supervision can increase the power imbalance between the aggressor and the victim. Based on L-RAT, in this study, we developed the SCOAC, defining cyberbullying (CYB) as intentional online aggression aimed at humiliating, intimidating, defaming, exposing, or excluding the victim. Cyberbullying can be repeated online through re-exposure due to the persistence, replicability, and publicity of one or more defamatory messages. Considering L-RAT, CYB occurs when these online activities expose a suitable target to contact with motivated offenders in environments with low protection or mediation (e.g., inadequate parental supervision), fostering an imbalance of power.

### 2.3 Adherence to test development guidelines

In this study, we adhered to recognized good practice protocols in the development and validation of psychological scales and tests, including the ITC Guidelines for Translating and Adapting Tests (17), the guidelines contained in the Standards for Educational and Psychological Testing manual (18), and the normative resolution of the Federal Council of Psychology of Brazil (nr° 31/2022), which establishes guidelines for conducting psychological assessments and regulates the psychological test evaluation system in Brazil (19).

### 2.4 Content and face validity

We invited four experts (judges) to evaluate the SCOAC using a Likert scale from 1 to 5 across three criteria: clarity of language, practical relevance, and the dimension of each item. The judges’ feedback was important because it helped refine the questionnaire instruments; their responses allowed improvements in grammar, especially in the cohesion of the questions, before conducting a pilot study. Each judge held a PhD in Health Psychology, specializing in bullying or cyberbullying. Significantly, the Likert scale was recoded as a binary variable (yes/no), with responses 1–3 indicating unclear or practically irrelevant, and responses 4–5 indicating clear or relevant.

We calculated the inter-judge agreement index based on the criteria of language clarity, practical relevance, and the specific dimension for each item. The agreement coefficient was determined using Fleiss’ Kappa coefficient. Values closer to 1 indicate higher agreement among raters, with the following interpretations: 0.01–0.20 (very low agreement), 0.21–0.40 (low agreement), 0.41–0.60 (moderate agreement), 0.61–0.80 (high agreement), and 0.81–1.00 (very high or perfect agreement).

### 2.5 Pilot test

We conducted a pilot test with seven adolescents (ages 13-17) from a focus group to evaluate the clarity and relevance of the items in the initial version of the SCOAC. The adolescents were recruited through the snowball method. For two hours, we discussed each item on the scale with them, gathering feedback on potential ambiguities, difficult terms, and the practical importance of the questions. Based on this feedback, we revised some questions to use a more youthful online vocabulary.

### 2.6 Measures

Sociodemographic data: We collect the following information: gender, age, grade level, and the highest education level of the father and mother.

#### 2.6.1 Florence cyber-aggression–cyber-victimization scales

This instrument comprises 14 questions and measures how often adolescents engage in or experience cyber-aggression and cyber-victimization, both in person and online. It evaluates behaviors commonly linked to cyberbullying, such as sending harmful messages, receiving or issuing threats online, and experiencing exclusion online, among others. A higher score indicates more severe behaviors in terms of frequency and intensity. In the Brazilian version of the scale (20), reliability coefficients were above 0.70 for all three factors.

#### 2.6.2 Internet addiction test

One of the most widely used tools for measuring internet use. It includes 20 Likert-type questions, scored from 0 to 100 points, with scores above 50 suggesting potential problematic internet use. The Brazilian version of the IAT (21) showed excellent internal consistency (α = 0.96).

#### 2.6.3 Problematic internet use questionnaire – short form

This tool was developed to identify problematic internet use; its short form contains 9 items, rated on a 5-point Likert scale. Scores range from 9 to 45, with higher scores indicating more severe symptoms. The Brazilian version of the scale (22) demonstrated excellent internal consistency (α = 0.91).

#### 2.6.4 Smartphone addiction scale – short version

This is one of the most widely used tools to measure smartphone dependence, as smartphones are among the most popular devices for internet use today. The 10-item SAS-SV and its Brazilian version (23) demonstrated high internal consistency (α = 0.82).

#### 2.6.5 Smartphone addiction inventory

This scale measures different patterns of smartphone use with 10 items answered by yes/no responses. It assesses smartphone use across the following dimensions: Compulsive Behavior, Preoccupation, Uncontrolled Use, Withdrawal, Social Dependence, and Environmental Influence. The SPAI-SF was translated and adapted in Brazil (24) with good internal consistency (α = 0.88).

#### 2.6.6 Self-perception of text-message dependency scale

It measures texting behavior, regardless of the device used. The STDS is especially useful because it helps identify texting patterns, which are a key aspect of cyberbullying. It was created using questions that assess adolescents’ need to check and reply to messages constantly. The higher the score, the more severe and intense the symptoms. The Brazilian version of the STDS (25) demonstrated high internal consistency (α = 0.89).

#### 2.6.7 Depression anxiety stress scale

It has 21 items in total, each assessing anxiety, depression, and stress. The DASS-21 is a quick-to-administer scale that has been adapted and validated for use with the Brazilian population (26). The dimensions demonstrated internal consistency ranging from 0.83 to 0.90. In this study, we did not use cutoff points; instead, we used the raw scores for each dimension.

#### 2.6.8 Impulsive behavior scale – short version

This tool has 20 items with a total score ranging from 20 to 80. Higher scores indicate greater impulsivity and compulsivity. The UPPS-P measures impulsivity across five specific dimensions: urgency, perseverance, premeditation, sensitivity to reward, and sensitivity to punishment. The Brazilian version (27) shows high internal consistency (α = 0.90).

#### 2.6.9 Difficulties in emotion regulation scale

It includes 18 items and primarily aims to assess six key areas related to emotion regulation: acceptance of emotional responses, the ability to pursue goals during intense emotions, impulsivity in response to strong feelings, emotional awareness, the capacity to sustain goal-oriented behaviors under emotional stress, and access to effective emotion regulation strategies. The Brazilian version (28) demonstrated excellent internal consistency (α = 0.94).

#### 2.6.10 Questionnaire for aggressive and reactive peer behaviors

This scale measures a variety of aggressive behaviors adolescents display toward their peers during interactions, whether at school or outside of school (29). The Q-CARP uses a Likert-type scale from 1 (“never”) to 4 (“every day”), with higher scores indicating greater aggression.

#### 2.6.11 Kidscreen-10

It evaluates quality of life in children and adolescents through 10 questions covering five areas: physical well-being, psychological well-being, autonomy and self-determination, and school environment. Items are rated on a scale of 1 to 5, from “never” to “always,” with a total score ranging from 10 to 50. Higher scores reflect a better quality of life. In the Brazilian version (30), the internal consistency of the items ranged from 0.65 to 0.88.

#### 2.6.12 Craft/cesare

This scale has six items answered with yes/no and was designed to identify risk patterns and effects of psychoactive substance use among adolescents. The scale was translated and adapted for use in Brazil (31) and demonstrated moderate internal consistency (α = 0.78).

### 2.7 Data analysis

#### 2.7.1 Internal structure and reliability

To evaluate the internal structure of the SCOAC, we performed a cross-validation process by randomly splitting the sample into two parts: 60% (n = 385) for Exploratory Factor Analysis (EFA) and 40% (n = 257) for Confirmatory Factor Analysis (CFA). The split was performed in Microsoft Excel^®^, using the “=RAND()” function, which generates values between 0 and 1. We used this procedure to ensure greater rigor in the internal structure assessment process, enabling the initial identification of the instrument’s dimensions in a subsample, with the refined structure then tested in another independent sample. One benefit of this split-data approach in factor analysis is that it prevents using the same sample to suggest and validate the structure, as the exploratory data are tested on an independent subsample, thereby reducing the risk of overfitting. After assigning random values to each participant, the cases were ordered and divided into two groups according to a 60/40 ratio.

For EFA, we applied the Weighted Least Squares (WLS) extraction method, which is appropriate for ordinal data, along with polychoric/tetrachoric correlation matrices, yielding more accurate estimates of relationships among ordinal categorical variables. The number of factors was determined using parallel analysis with oblique promax rotation. The adequacy of the analysis was previously confirmed using the Kaiser-Meyer-Olkin (KMO) index and Bartlett’s test of sphericity, both of which were statistically significant.

In addition to EFA, we conducted a Network Analysis (NA) as a second, complementary exploratory step to examine the scale’s psychometric properties. The NA was carried out using the LASSO method based on partial correlations to identify how the items are organized within the network. The importance of each item was evaluated with four centrality measures: strength (the strength of connections an item has with others), betweenness (an item’s ability to act as a link between different items), closeness (how close an item is to others), and expected influence (the overall impact of the item in the network, considering both positive and negative connections). Items with low centrality scores were excluded based on these indicators. The final selection combined EFA (factor loadings) and NA (centrality) criteria before performing Confirmatory Factor Analysis.

For CFA, we used the ULSM estimator, which is well-suited to ordinal items. The choice also considered preliminary analyses with alternative estimators, which yielded less stable standardized solutions in parts of the model, including factor loadings above 1 for some items, which led us to adopt a more conservative and empirically stable confirmatory specification. We also conducted a Multigroup Confirmatory Factor Analysis (MGCFA) to assess invariance across sex (boys vs. girls), sequentially testing the configural, metric, scalar, and strict models. The model was considered adequate when invariance reduction did not exceed 0.02, and the increase in RMSEA remained below 0.015 (32). The fit was deemed satisfactory when the adjustment parameters showed TLI and CFI ≥ 0.95, a χ²/df ratio ≤ 3, RMSEA ≤ 0.08, and SRMR ≤ 0.05, as per the literature (32). For internal consistency, we used Cronbach’s alpha (α) and McDonald’s omega (ω); the latter is more appropriate when tau-equivalence is not satisfied because it accounts for differences in factor loadings and variances among items (33). We also examined item performance using the alpha-if-item-dropped and omega-if-item-dropped indices (34).

All structural analyses, such as EFA, CFA, and network analysis, were performed using JASP software (version 0.95.4.0), an open-source statistical tool.

#### 2.7.2 Criterion validity

We evaluated the SCOAC items’ ability to correlate with another measure of the same construct, cyberbullying, as evidence of criterion validity. To do this, we computed Spearman correlations between the SCOAC score and its dimensions, comparing its performance against the total score of the Florence Cyber Aggression–Cyber Victimization Scales (FCBVSs), focusing only on the 9 victimization-related items. We selected the FCBVSs because they are the only instrument validated in Brazil that specifically assesses cyberbullying behaviors. In this study, we considered Spearman correlations of 0.50 or higher as adequate evidence of criterion validity, in line with recommendations from the psychometric literature (35).

#### 2.7.3 Convergent validity

The convergent validity of the SCOAC was evaluated using the SPAI-SF, SAS-SV, STDS, IAT, and PIUQ-9 instruments. The total scores of these instruments were correlated with the overall SCOAC score and its subscales using Spearman’s correlation coefficients. In this study, we regarded moderate correlations (≥ 0.30) as indicative of adequate convergent validity (35). Unlike criterion-related validity, which predicts stronger correlations because it measures the same construct, convergent validity permits moderate correlations since it involves related but not identical constructs.

#### 2.7.4 Nomological validity

We evaluated how SCOAC scores relate to other measures that assess constructs technically linked to cyberbullying, such as emotional vulnerability, depression, anxiety, stress, and others. We aimed to determine whether these scores are interconnected within a broader theoretical context that extends beyond cyberbullying. To achieve this, we employed the following instruments: Q-CARP Scale, Aggressive Behavior Scale (ECA), Reactive Behavior Scale (ECR), Depression (DASS-21), Anxiety (DASS-21), Stress (DASS-21), DERS-18, UPPS-P, KIDSCREEN, and CRAFT/CESARE.

#### 2.7.5 Predictive validity

To evaluate the predictive validity of the SCOAC, we performed an ROC curve analysis to assess the scale’s accuracy in detecting cyberbullying behaviors, using only the victimization items (9 questions) from the Florence Cyber Aggression-Cyber Victimization Scale (FCBVSs) as the reference. Since the FCBVSs lack an established cutoff point, we initially conducted a Latent Profile Analysis (LPA) to categorize participants into distinct risk profiles. In the LPA, common fit indicators were examined in latent class models (log-likelihood, AIC, cAIC, BIC, aBIC, and entropy). Although the three-class model provided slightly better fit indices, we chose the two-class model based on the principle of parsimony, given its fewer variables and clearer interpretability. **Table 1** displays the fit indices for one of the criteria used.

**Table 1:** Criteria for selecting the best fit index of the latent class analysis (LCA) model.

Classes	Log-likelihood	AIC	cAIC	BIC	aBIC	Entropy
1	-1247,3	2512,6	2561,8	2552,8	2524,2	–
**2**	-1031,9	2101,7	2205,6	2186,6	2126,2	0,796
3	-979,0	2015,9	2174,4	2145,4	2053,3	0,854
4	-966,3	2010,5	2223,6	2184,6	2060,8	0,841
5	-957,1	2012,2	2280,0	2231,0	2075,4	0,872
6	-951,0	2020,1	2342,5	2283,5	2096,2	0,802

Log-likelihood, Log-verosimilitude; AIC, Akaike Information Criterion; cAIC, Consistent Akaike Information Criterion; BIC, Bayesian Information Criterion; aBIC, Adjusted Bayesian Information Criterion.

**Figure 1:**
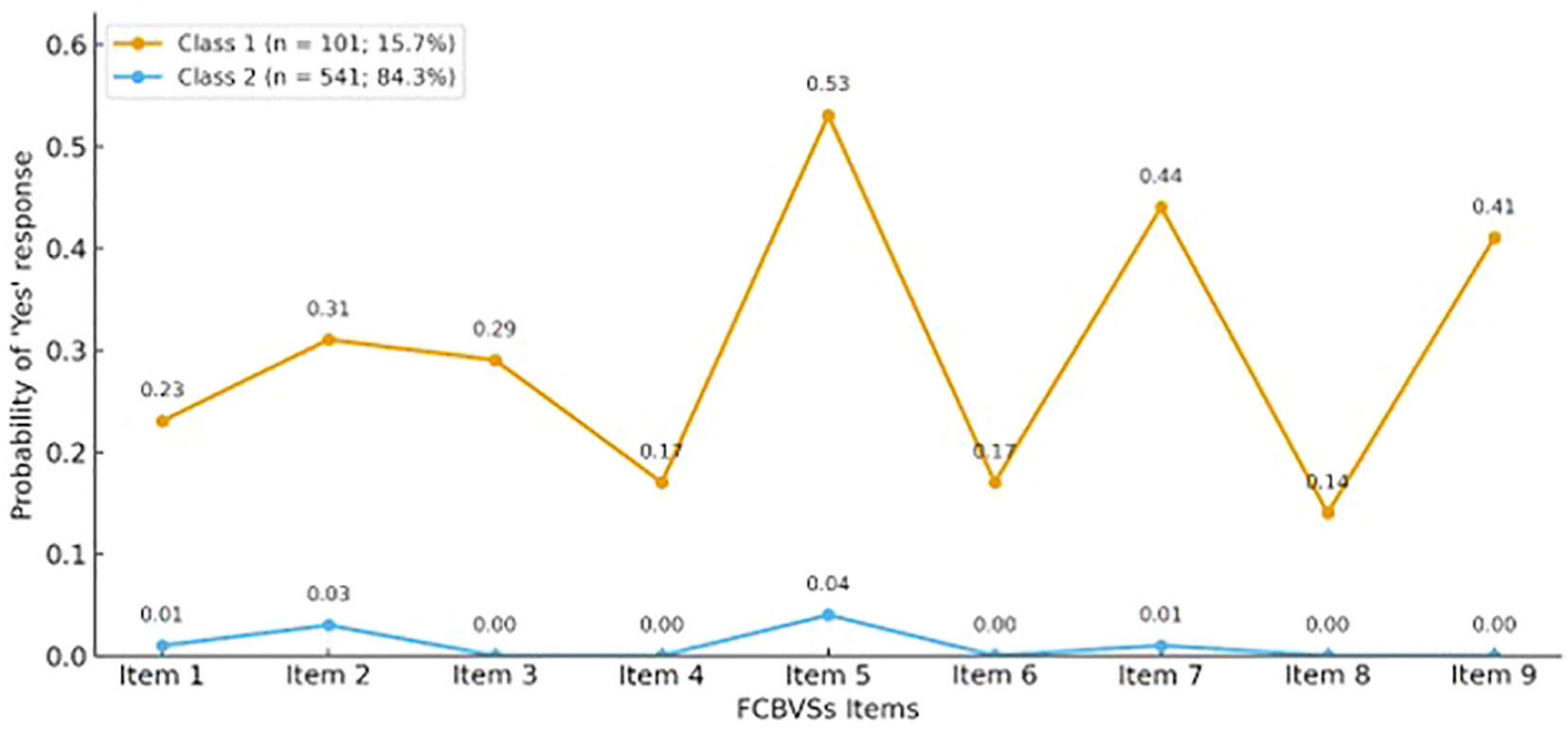
Latent class profiles of the florence cyberbullying-cybervictimization scale (FCBVSs).

Therefore, Profile 1 was identified as a group with a higher likelihood of involvement in cyberbullying (cases/positives). In comparison, Profile 2 was recognized as a group with a lower likelihood (non-cases/negatives), as shown in **Figure 1**. This classification served as a reference for constructing the ROC curve.

The ROC curve analysis identified various sensitivity-specificity combinations to determine cutoff points for SCOAC. We initially used the Youden index (J) to balance these aspects; however, we also applied the decision-theoretic approach proposed by Smits et al. (36), which accounts for the costs of both correct and incorrect classifications. Consequently, we placed greater emphasis on false negatives, given their clinical and social importance, rather than on false positives, following the work of other researchers (37). The relative costs considered were: false positives = 0.03, false negatives = 1.0, true positives = 0.89, and true negatives = 0.001.

#### 2.7.6 Psychosocial profile

After conducting latent profile and ROC analyses, we compared the groups classified as Cyberbullying Risk (CYB) and Non-Cyberbullying Risk (nCYB) to determine whether the screening classification based on SCOAC scores was associated with psychological and psychiatric characteristics related to cyberbullying. This step aimed to evaluate the instrument’s practical and discriminative usefulness beyond its internal structure, particularly its ability to identify adolescents at greater risk for emotional and behavioral challenges.

## 3 Results

Of the total participants (N = 642), 51.4% were boys (n = 330) and 48.6% were girls (n = 312), aged 11–17 years (M = 13.4; SD = 1.78). Most participants were in elementary school (n = 517; 80.6%), while 125 adolescents (19.4%) were in high school. Regarding paternal education, among adolescents who could provide information, 19.2% (n = 123) reported completing high school, 17.6% (n = 113) completing higher education, 13.9% (n = 89) completing postgraduate studies, and 11.5% (n = 74) completing only elementary education. For mothers, the distribution was similar, although with slightly higher levels: 24.0% (n = 154) had completed high school, 19.3% (n = 124) had higher education, 17.4% (n = 112) had postgraduate education, and 7.5% (n = 48) had completed elementary education.

### 3.1 Internal structure (exploratory models)

Initially, we performed an EFA with the 30 initial items of the SCOAC scale, but the overall KMO index was very low (0.224), with similarly low individual values. Although Bartlett’s test of sphericity was significant (χ² = 19316.79; *p <* 0.001), the fit indices indicated poor model quality: RMSEA = 0.309, TLI = 0.144, and CFI = 0.475, all of which fell outside the acceptable ranges. Promax rotation revealed six factors that explained 60.5% of the variance, but with modest factor loadings and overlap among them.

Due to the initial results showing an unsatisfactory factor structure, we adjusted the model. We retested the EFA with stricter criteria for item retention in the final SCOAC version (see **Table 2**). We excluded items with factor loadings below 0.50 (9 items) and those with low centrality indices in the network analysis (4 items). Based on these criteria, the SCOAC now comprises 17 items, and the new EFA model exhibits more robust psychometric indices: the overall KMO is excellent (0.890), and Bartlett’s test of sphericity is significant (χ² = 5121.481; *p <* 0.001).

**Table 2:** Descriptive statistics, factor loadings, and reliability indices (Cronbach’s α, McDonald’s ω, and item-dropped analyses) for SCOAC items and factors from exploratory factor analysis (EFA) using 60% of the sample (n=385).

Factors/Questions	Item	*M*	*SD*	Factorial loading	Cronbach's α	McDonald's ω	α if item dropped	ω if item dropped
Threat					0.84	0.86		
Someone made fun of me, insulted me, or called me names online.	1	0.69	1.28	0.62			0.84	0.85
I felt threatened or unsafe because of something that happened online.	2	0.35	0.89	0.65			0.80	0.83
Someone threatened to hurt me physically on the internet.	3	0.33	0.91	0.69			0.81	0.83
Someone threatened to spread lies about me online.	4	0.38	0.90	0.67			0.81	0.83
Someone threatened to exclude me from something online.	5	0.35	0.88	0.65			0.83	0.85
Someone said they would get back at me or take revenge on me online.	6	0.26	0.72	0.64			0.82	0.83
Defamation / Exposure					0.83	0.84		
Someone shared a photo or video of me without my permission.	7	0.58	1.10	0.73			0.80	0.81
Someone gave me a mean or nasty nickname online.	8	0.51	1.12	0.50			0.80	0.80
Someone made a meme or edited a picture about me.	9	0.53	1.05	0.78			0.82	0.82
Someone posted a screenshot of my private conversation without asking me.	10	0.74	1.29	0.65			0.80	0.81
Someone shared my personal information (such as my address or phone number) without my permission.	11	0.36	0.96	0.55			0.81	0.81
Someone told a secret or shared something private I said in messages or on social media.	12	0.47	1.02	0.58			0.80	0.81
Emotional Problems					0.87	0.87		
Being attacked online is messing with my mental and emotional health.	13	0.44	0.94	0.59			0.86	0.87
It has been hard to focus since I started getting mean messages or comments online.	14	0.36	0.86	0.75			0.83	0.84
It has been hard to sleep since I started getting mean messages or comments online.	15	0.33	0.87	0.83			0.85	0.85
It has been hard to do everyday things during the day since I started getting mean messages or comments online.	16	0.28	0.81	0.83			0.83	0.83
I have noticed changes in my mood or behavior since I started getting mean messages or comments online.	17	0.49	1.06	0.71			0.85	0.85

*M*, mean; *SD*, standard deviation. The overall Cronbach’s α was.915 and the overall McDonald’s ω was.919.

The overall internal consistency coefficients of SCOAC were Cronbach’s α = 0.915 and McDonald’s ω = 0.919.

Another highlight is that the three extracted factors together explained 72.4% of the total variance in the data. Compared with the initial 30-item solution, the refined 17-item version demonstrated significant improvements in various psychometric indicators, including higher KMO, reduced factor overlap, and a greater percentage of explained variance. Regarding fit indices, the SRMR was adequate, and CFI and TLI showed improvements over the original model. However, the RMSEA remained high, indicating caution when interpreting the exploratory solution on its own. Therefore, the framework was later tested on an independent subsample using CFA, which showed a more consistent overall fit. Correlations between the factors ranged from moderate to high (0.619-0.725), supporting the existence of interconnected dimensions.

**Figure 2:**
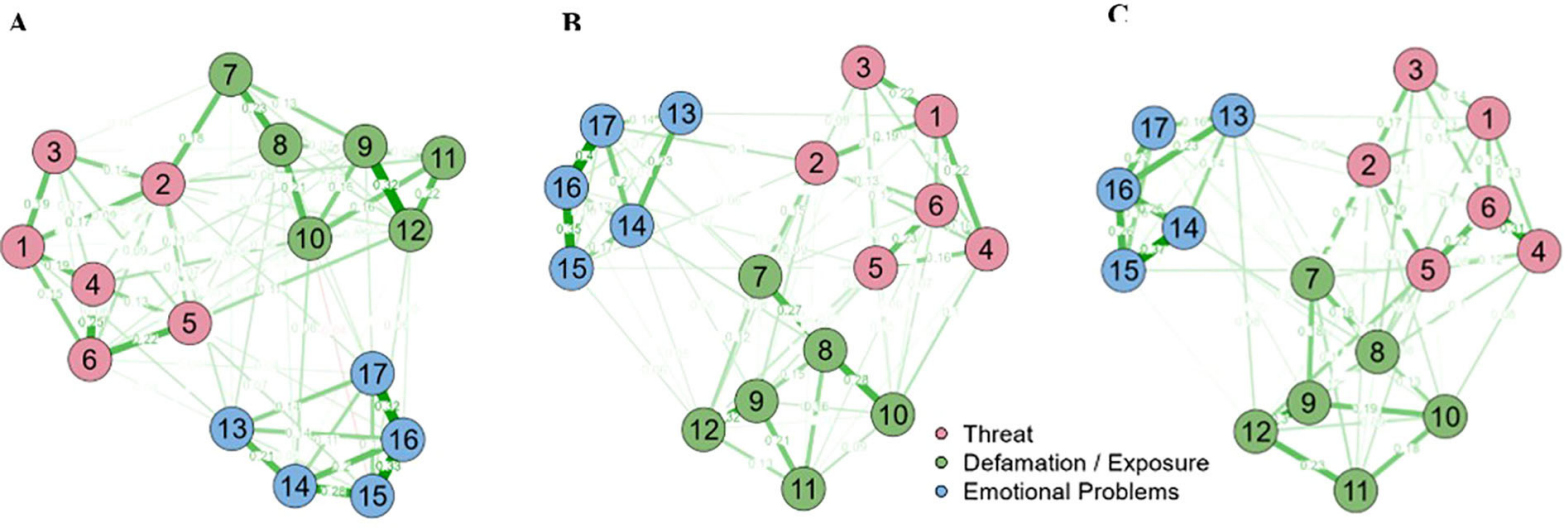
Gaussian graph model based on network analysis to examine the relationship between the 17 items of the SCOAC scale, considering: (i) the overall samle **(A)**; (ii) gender: girls **(B)**, boys **(C)**. True edges indicate positive correlations, while red edges show negative correlations. The stronger the correlations, the thicker the edges.

The Network Analysis (NA) model is illustrated in **Figure 2**, where a coherent distribution was observed among the SCOAC items across the three dimensions in its final version, encompassing all participants (**Figure 2A**). The highest correlations were observed within the Defamation/Exposure dimension, with thicker edges than in the other dimensions. Additionally, the NA model remained consistent when considering participants’ sex (boys vs girls), indicating strong psychometric qualities of the SCOAC (**Figures 2B, C**).

Regarding the centrality levels of the items in the network, across all participants (**Table 3**), the items with the highest centrality are primarily in the Threat and Defamation/Exposure dimensions. Within Threat, Item 6 (“I received some kind of threat of retaliation online”) showed high values for strength (1.217) and expected influence (1.370), making it among the most significant items in the system. In the Defamation/Exposure dimension, Item 8 (“Someone created a bad/mean nickname against me online”) also stood out, with the highest scores in closeness (1.876), strength (1.239), and expected influence (1.393). Regarding the Emotional Problems dimension, Item 16 (“I have difficulty carrying out daily activities since I started receiving offensive messages or comments online”) exhibited high centrality, especially in strength (1.448) and influence (1.396).

**Table 3:** Centrality measures for the 17 items of the SCOAC scale, based on a 60% sample (*n =* 385).

General sample (*n=* 385)				
Item/dimension	Betweenness	Closeness	Strength	Expected influence
Threat				
Item 1	0.259	0.554	-0.998	-0.969
Item 2	-0.370	0.514	0.493	0.625
Item 3	-0.580	0.480	0.349	0.477
Item 4	1.519	1.608	0.467	-0.134
Item 5	-1.420	-0.174	-1.960	-1.898
Item 6	0.259	0.335	1.217	1.370
Defamation / Exposure				
Item 7	0.469	0.334	-0.627	-0.527
Item 8	1.309	1.876	1.239	1.393
Item 9	-1.630	-1.299	-1.815	-1.748
Item 10	-0.580	-0.086	0.022	-0.071
Item 11	1.099	1.063	-0.414	-0.307
Item12	-1.210	-0.079	0.652	0.788
Emotional Problems				
Item 13	0.049	-0.825	-0.943	-0.852
Item 14	1.309	-0.526	0.345	0.413
Item 15	0.889	-0.839	0.396	-0.206
Item 16	-0.580	-1.340	1.448	1.396
Item 17	-0.790	-1.597	0.129	0.251

Positive values indicate greater influence of the item in the overall organization of the network.

The table presents the values of the betweenness, closeness, strength, and expected influence metrics, applied to all items and grouped into the scale’s three dimensions: Threat, Defamation/Exposure, and Emotional Problems.

When examining the levels of centrality related explicitly to sex (**Table 4**), public exposure (e.g., photos/videos without permission) was the most central item for girls. For boys, the main SCOAC items were explicit threats of exclusion and tangible challenges in daily activities after experiencing attacks.

**Table 4:** Centrality measures for the 17 items of the SCOAC scale, based on 60% of the sample (n = 385), are presented by sex (female/male) among adolescents.

Girls (*n=* 123)				
Item/dimension	Betweenness	Closeness	Strength	Expected influence
Threat				
Item 1	-0.454	1.045	-0.853	-0.853
Item 2	1.046	1.133	0.501	0.501
Item 3	-0.025	0.107	1.324	1.324
Item 4	-0.454	0.405	-0.078	-0.078
Item 5	-0.882	-1.015	-1.292	-1.292
Item 6	-0.775	-0.186	0.409	0.409
Defamation / Exposure				
Item 7	0.832	0.762	0.088	0.088
Item 8	2.331	2.490	1.242	1.242
Item 9	-0.882	-1.213	-1.880	-1.880
Item 10	-0.346	0.030	0.220	0.220
Item 11	-0.882	-0.830	-0.971	-0.971
Item12	-0.346	0.190	0.714	0.714
Emotional Problems				
Item 13	-0.882	-1.369	-1.459	-1.459
Item 14	0.403	-0.437	0.138	0.138
Item 15	-0.775	-0.994	-0.335	-0.335
Item 16	0.189	-0.476	0.906	0.906
Item 17	1.902	0.358	1.325	1.325
Boys (*n=* 134)				
Item/dimension	Betweenness	Closeness	Strength	Expected influence
Threat				
Item 1	-0.490	0.639	-0.393	-0.393
Item 2	0.700	1.278	0.543	0.543
Item 3	-0.192	0.606	-0.163	-0.163
Item 4	-0.936	-0.206	-0.433	-0.433
Item 5	-1.085	-0.547	-1.643	-1.643
Item 6	1.443	1.258	1.520	1.520
Defamation / Exposure				
Item 7	-0.936	-0.674	-1.355	-1.355
Item 8	0.997	1.498	0.567	0.567
Item 9	-1.085	-0.574	-0.871	-0.871
Item 10	-0.639	-0.421	-0.329	-0.329
Item 11	1.295	1.611	1.000	1.000
Item12	0.402	0.300	0.601	0.601
Emotional Problems				
Item 13	1.741	-0.158	0.404	0.404
Item 14	-0.936	-1.297	-0.072	-0.072
Item 15	-0.044	-1.227	0.482	0.482
Item 16	0.849	-0.605	1.733	1.733
Item 17	-1.085	-1.482	-1.594	-1.594

Positive values indicate greater influence of the item in the overall organization of the network.

The table shows the values of betweenness, closeness, strength, and expected influence metrics, applied to all items and grouped into the scale’s three dimensions: Threat, Defamation/Exposure, and Emotional Problems.

### 3.2 Internal structure (confirmatory models)

**Figure 3:**
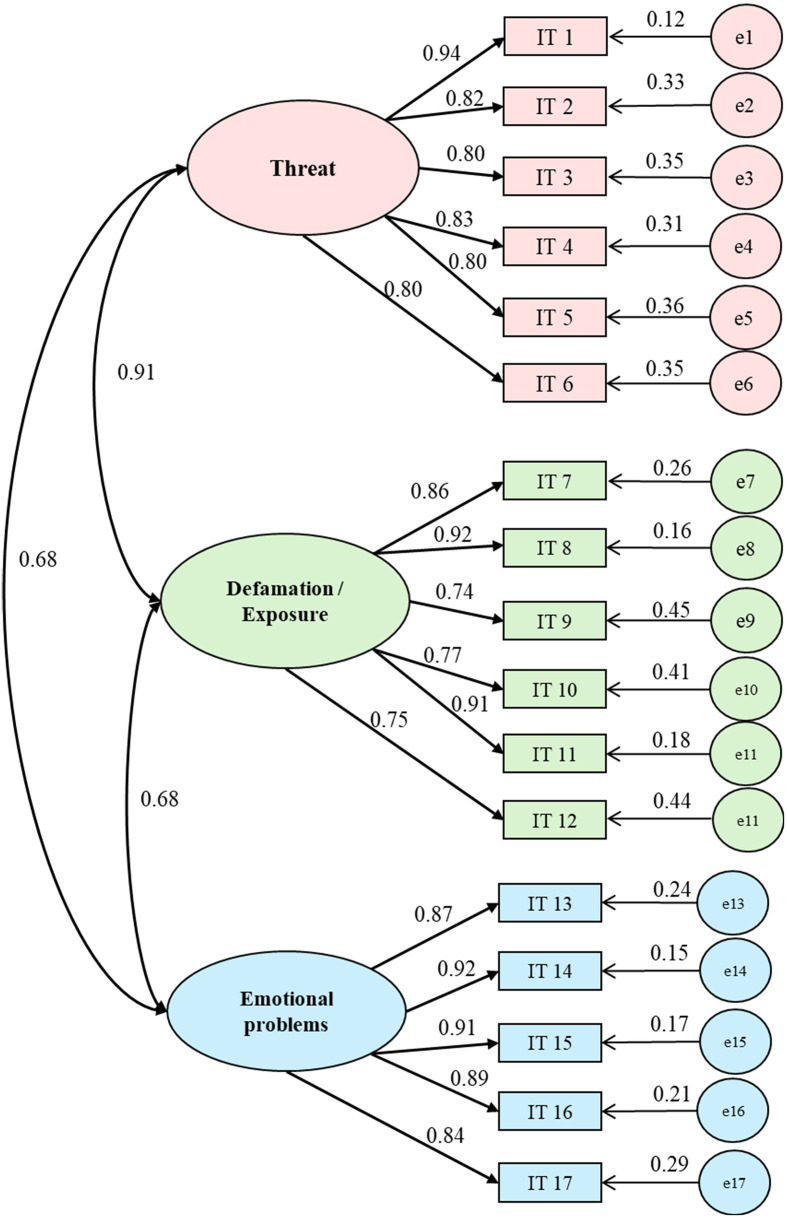
Confirmatory Factor Analysis of the SCOAC three-factor model (Threat, Defamation/Exposure and Emotional Problems). The model demonstrated excellent fit indices (CF1 = 0.991, TLI = 0.989, RMSEA = 0.030, SRMR = 0.048), considering the three-factor structure of the scale.

The three-factor, 17-item SCOAC solution was evaluated using a CFI, as illustrated in the factor diagram (**Figure 3**). Overall, the results indicate a good fit for this model: χ²(116) = 142.29; RMSEA = 0.002–0.045; SRMR = 0.048; TLI = 0.989; CFI = 0.991.

We also conducted an MGCFA concerning sex (**Table 5**), and all levels of invariance (configural, metric, scalar, and strict) showed good fits (CFI ≥.97; low RMSEA). In this regard, the SCOAC demonstrated invariance between boys and girls, indicating that its structure is consistent across groups and that the scale can be confidently applied to both groups.

**Table 5:** Confirmatory factor analyses (CFI) of the SCOAC and multigroup confirmatory factor analysis (MGCFA) fit indexes for sex, based on 40% of the sample (n= 257).

Model	Goodness-of-fit indexes					
	χ²	df	RMSEA (90% CI)	TLI	SRMR	CFI
Overall model (CFI)	142.29	116	(0.002 - 0.045)	0.989	0.048	0.991
MGCFA model						
Male (*n=* 134)	130.10	116	(0.000 - 0.055)	0.988	0.066	0.990
Female (*n=* 123)	130.13	116	(0.000 - 0.057)	0.990	0.062	0.991
*Configural Model*	260.10	232	(0.000 - 0.049)	0.989	0.064	0.991
*Metric Model*	253.66	246	(0.000 - 0.040)	0.977	0.081	0.979
*Scalar Model*	267.80	260	(0.000 - 0.039)	0.978	0.082	0.979
*Strict Model*	280.96	277	(0.000 - 0.037)	0.989	0.094	0.989

*χ²*, chi-square statistic; *df*, degrees of freedom; RMSEA, Root Mean Square Error of Approximation (90% confidence interval), with values ≤.06 indicating good fit; TLI, Tucker–Lewis Index, with values ≥.95 indicating good fit; SRMR, Standardized Root Mean Square Residual, with values ≤.08 indicating good fit; CFI, Comparative Fit Index, with values ≥.95 indicating good fit.

### 3.3 Reliability

Regarding internal consistency, the SCOAC demonstrated excellent overall reliability (α = 0.915, ω = 0.919). For its dimensions, the Threat factor showed α = 0.848 and ω = 0.863; the Defamation/Exposure factor had α = 0.837 and ω = 0.840; and the Emotional Problems factor exhibited the strongest internal consistency, with α = 0.873 and ω = 0.879.

### 3.4 Criterion, convergent, and nomological validities

Based on Spearman’s correlations, we observed strong internal correlations among the three factors of the SCOAC (**Table 6**). The total SCOAC scores showed strong correlations with Threat (ρ = 0.796), Defamation/Exposure (ρ = 0.845), and Emotional Problems (ρ = 0.714), indicating that these dimensions are well-integrated into the overall construct. Regarding criterion validity, the correlations with the Florence Victimization Scale were all significant. They ranged from moderate to substantial, with the total Florence score correlating with the total SCOAC score (ρ = 0.649).

**Table 6:** Spearman correlation coefficients for the total sample (n= 642), showing the total and factor scores of the SCOAC to evaluate three types of validity: criterion, convergent, and nomological.

Variables	SCOAC (*n=* 642)			
	Total score *(ρ)*	Threat	Defamation / exposure	Emotional problems
SCOAC Factors				
Threat	0.796***	–	–	–
Defamation / Exposure	0.845***	0.605***	–	–
Emotional Problems	0.714***	0.469***	0.449***	–
	Criterion Validity			
Florence Scale – Victimization				
Visual dimension	0.485***	0.415***	0.454***	0.350***
Falsification dimension	0.501***	0.433***	0.475***	0.404***
Exclusion dimension	0.619***	0.480***	0.583***	0.465***
Total score	0.649***	0.539***	0.601***	0.470***
	Convergent Validity			
Total scores				
SPAI-SF	0.350***	0.275***	0.301***	0.284***
SAS-SV	0.356***	0.247***	0.285***	0.320***
STDS	0.272***	0.193***	0.237***	0.245***
IAT	0.321***	0.277***	0.228***	0.297***
PIUQ-9	0.296***	0.217***	0.220***	0.290***
Total scores	Nomological Validity			
Aggressiveness / Interpersonal Behaviors				
Q-CARP Scale (Peer Aggressiveness Questionnaire)	0.334***	0.324***	0.300***	0.245***
ECA – Aggressive Behaviors Scale	0.304***	0.277***	0.298***	0.189***
ECR – Reactive Behaviors Scale	0.319***	0.321***	0.275***	0.252***
Emotional Distress – DASS-21				
Depression	0.387***	0.289***	0.313***	0.327***
Anxiety	0.437***	0.329***	0.358***	0.359***
Stress	0.404***	0.304***	0.337***	0.320***
Self-Regulation and Psychological Functioning				
DERS-18	0.368***	0.293***	0.281***	0.339***
UPPS-P	0.156***	0.146***	0.119***	0.125**
KIDSCREEN	-0.103**	-0.064	-0.082*	-0.118***
CRAFT/CESARE	0.277***	0.233***	0.279***	0.241***

*ρ,* Spearman correlation symbol (rho), *p < 0,05, **p < 0.01, ***p < 0.001.

Regarding convergent validity, the SCOAC total score showed stronger correlations with instruments related to smartphone use (SPAI-SF and SAS-SV, although correlations with other instruments were also statistically significant. Concerning nomological validity, we observed that the strongest correlations of the SCOAC total scores were with measures of emotional distress, especially anxiety (ρ = 0.437) and stress (ρ = 0.404), followed by depression (ρ = 0.387). Additionally, significant correlations were found with emotional dysregulation (ρ = 0.368) and peer aggression (ρ = 0.334). Regarding impulsivity, a significant but weaker correlation was observed (ρ = 0.156).

### 3.5 Predictive validity

ROC curve analysis showed that the SCOAC had an area under the curve (AUC) of 0.85 (95% CI: 0.82–0.88; SE = 0.021; z = 15.98; p < 0.0001), indicating excellent ability to distinguish between case and control groups (**Table 7**). The most suitable cutoff point, identified by the Youden index (J = 0.56), was≥8, with a sensitivity of 77.2% and a specificity of 78.9%, providing the best balance between these measures. To generate the ROC curve, we used the LPA model’s classification as the reference, which identified 15.7% of cases. Applying a cutoff of ≥8 directly to the sample yielded a prevalence of 29.9% among cases classified by the SCOAC.

**Table 7:** Receiver operating characteristic (ROC) analysis to identify the optimal cutoff score of the SCOAC for predicting cyberbullying, based on the latent profile derived from the nine victimization items of the florence cyber victimization scale.

Criterion	Sensitivity	95% CI	Specificity	95% CI	+LR	-LR
>0	94.06	87.5 - 97.8	36.78	32.7 - 41.0	1.49	0.16
>1	93.07	86.2 - 97.2	46.77	42.5 - 51.1	1.75	0.15
>2	92.08	85.0 - 96.5	54.53	50.2 - 58.8	2.02	0.15
>3	92.08	85.0 - 96.5	60.26	56.0 - 64.4	2.32	0.13
>4	90.10	82.5 - 95.1	65.25	61.1 - 69.3	2.59	0.15
>5	84.16	75.6 - 90.7	70.43	66.4 - 74.2	2.85	0.22
>6	82.18	73.3 - 89.1	73.20	69.3 - 76.9	3.07	0.24
>7	79.21	70.0 - 86.6	75.79	71.9 - 79.3	3.27	0.27
**>8**	**77.23**	**67.8 - 85.0**	**78.93**	**75.2 - 82.3**	**3.66**	**0.29**
>9	72.28	62.5 - 80.7	81.15	77.6 - 84.4	3.83	0.34
>10	69.31	59.3 - 78.1	84.29	80.9 - 87.3	4.41	0.36
>11	67.33	57.3 - 76.3	86.88	83.7 - 89.6	5.13	0.38
>12	66.34	56.2 - 75.4	89.28	86.4 - 91.8	6.19	0.38
>13	63.37	53.2 - 72.7	90.39	87.6 - 92.7	6.59	0.41
>14	60.40	50.2 - 70.0	91.31	88.6 - 93.5	6.95	0.43
>15	57.43	47.2 - 67.2	92.05	89.4 - 94.2	7.22	0.46
>16	56.44	46.2 - 66.3	92.98	90.5 - 95.0	8.03	0.47
>17	56.44	46.2 - 66.3	94.09	91.8 - 95.9	9.54	0.46
>18	52.48	42.3 - 62.5	94.64	92.4 - 96.4	9.79	0.50
>19	48.51	38.4 - 58.7	94.82	92.6 - 96.5	9.37	0.54
>20	46.53	36.5 - 56.7	94.82	92.6 - 96.5	8.99	0.56
>21	43.56	33.7 - 53.8	95.01	92.8 - 96.7	8.73	0.59
>22	40.59	30.9 - 50.8	95.19	93.0 - 96.8	8.45	0.62
>23	38.61	29.1 - 48.8	95.38	93.3 - 97.0	8.36	0.64
>24	36.63	27.3 - 46.8	95.56	93.5 - 97.1	8.26	0.66
>25	35.64	26.4 - 45.8	95.75	93.7 - 97.3	8.38	0.67
>26	32.67	23.7 - 42.7	95.93	93.9 - 97.4	8.03	0.70
>27	30.69	21.9 - 40.7	96.30	94.3 - 97.7	8.30	0.72
>28	27.72	19.3 - 37.5	96.67	94.8 - 98.0	8.33	0.75
>32	20.79	13.4 - 30.0	96.67	94.8 - 98.0	6.25	0.82
>33	20.79	13.4 - 30.0	97.41	95.7 - 98.6	8.03	0.81
>34	18.81	11.7 - 27.8	97.60	95.9 - 98.7	7.83	0.83
>35	16.83	10.1 - 25.6	97.78	96.2 - 98.8	7.59	0.85
>36	14.85	8.6 - 23.3	97.97	96.4 - 99.0	7.30	0.87
>37	12.87	7.0 - 21.0	98.34	96.9 - 99.2	7.74	0.89
>38	8.91	4.2 - 16.2	98.52	97.1 - 99.4	6.03	0.92
>39	6.93	2.8 - 13.8	98.71	97.4 - 99.5	5.36	0.94
>41	5.94	2.2 - 12.5	98.71	97.4 - 99.5	4.59	0.95
>42	5.94	2.2 - 12.5	98.89	97.6 - 99.6	5.36	0.95
>43	3.96	1.1 - 9.8	99.26	98.1 - 99.8	5.36	0.97
>44	3.96	1.1 - 9.8	99.45	98.4 - 99.9	7.14	0.97
>46	1.98	0.2 - 7.0	99.45	98.4 - 99.9	3.57	0.99
>47	1.98	0.2 - 7.0	99.63	98.7 - 100.0	5.36	0.98
>52	0.99	0.03 - 5.4	99.63	98.7 - 100.0	2.68	0.99
>56	0.99	0.03 - 5.4	100.00	99.3 - 100.0		0.99
>59	0.00	0.0 - 3.6	100.00	99.3 - 100.0		1.00

Criterion, SCOAC cutoff point; Sensitivity, proportion of true positives correctly identified; Specificity, proportion of true negatives correctly identified; 95% CI, 95% confidence interval; +LR, positive likelihood ratio; −LR, negative likelihood ratio.

The bold lines in the figures indicate the most accurate SCOAC score cutoffs for predicting cyberbullying cases.

**Figure 4:**
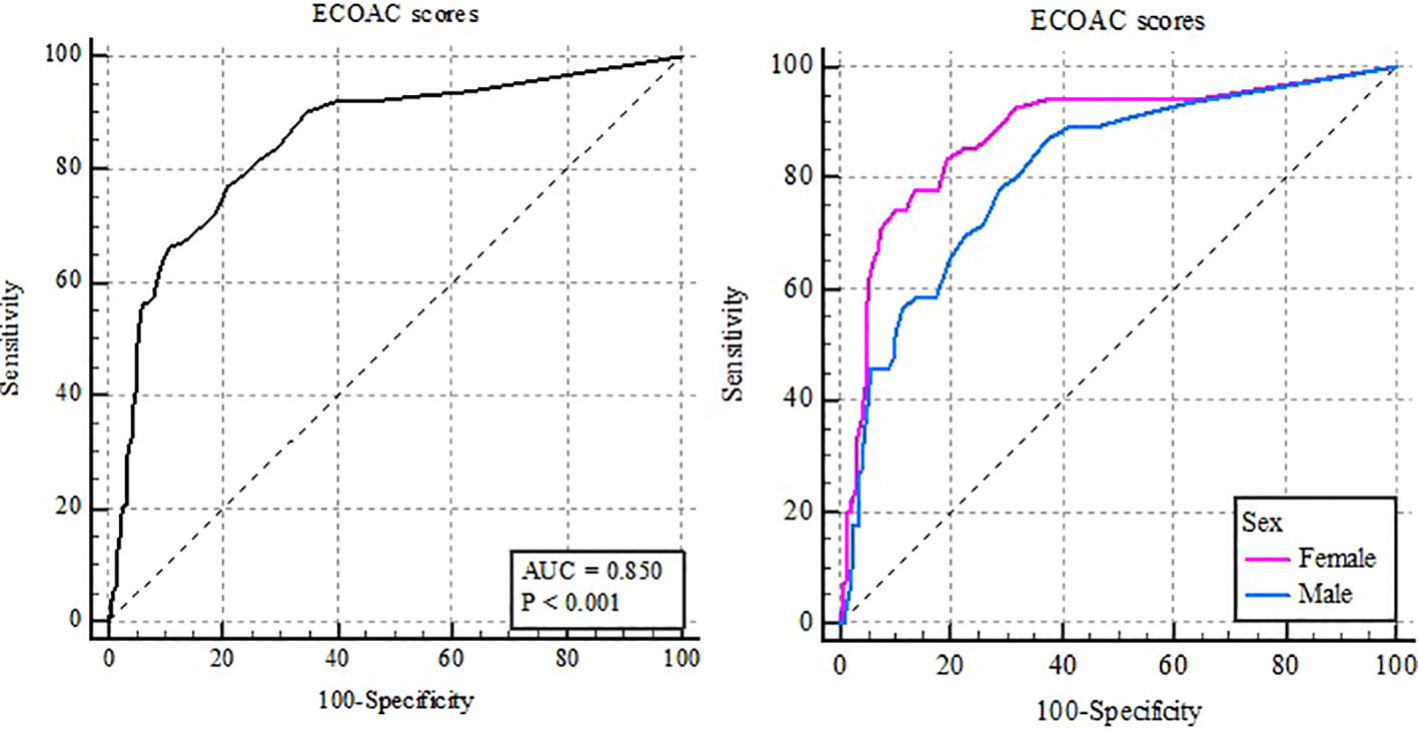
ROC curve analysis of the SCOAC score for predicting cyberbullying in the overall sample and by sex.

**Figure 4** displays the analysis separated by sex, with the AUC slightly higher among girls (AUC = 0.88; 95% CI: 0.84–0.91; SE = 0.027) than among boys (AUC = 0.81; 95% CI: 0.77–0.85; SE = 0.034). However, this difference was not statistically significant (p = 0.123), suggesting that the scale’s accuracy is similar for both boys and girls. Therefore, we recommend that a score≥ 8 serve as a valid cutoff for both sexes, supporting the instrument’s consistency.

### 3.6 Psychosocial profile of cyberbullying risk and non-cyberbullying risk participants

We found that 29.9% (n = 192) were classified in the CYB group, while the remaining adolescents (70.1%) were in the nCYB group (n = 450). Regarding sociodemographic characteristics, no significant differences were found between the groups (**Table 8**).

**Table 8:** Sociodemographic data of adolescents categorized as cyberbullying risk participants (CBY group; 29.9%; n = 192) and non-cyberbullying risk participants (nCBY group; 70.1%; n = 450).

Sociodemographic variables	CBY		nCBY		χ²	*p*
	*n*	*%*	*n*	*%*		
Sex					0.21	0.64
Female	96	50.0	216	48.0		
Male	96	50.0	234	52.0		
Father’s education level					0.87	0.83
Do not know	71	37.0	172	38.2		
Elementary school	21	10.9	53	11.8		
High school	41	21.4	82	18.2		
College/University degree	59	30.7	143	31.8		
Mother’s education level					3.86	0.27
Do not know	56	29.2	148	32.9		
Elementary school	17	8.9	31	6.9		
High school	54	28.1	100	22.2		
College/University degree	65	33.9	171	38.0		
School year					0.49	0.48
Middle school	152	79.2	367	81.6		
High school	40	20.8	83	18.4		

*n,* total frequency; %, percentage; *M,* mean; χ², chi-square test; *p,* significance level.

On the other hand, for various online activities (**Table 9**), the CYB group showed significantly higher average scores, particularly for smartphone use. When considering online activities and aggressive behavior patterns (**Table 10**), we observed the same trend, with the average scores being significantly higher in the CYB group. Additionally, this group exhibited greater severity of emotional problems, such as anxiety, depression, and stress, along with higher levels of impulsivity and emotional dysregulation, and lower levels of quality of life compared to the nCYB group (**Table 11**).

**Table 9:** Profile of various online activities among adolescents classified as cyberbullying risk participants (CBY group; 29.9%; *n =* 192) and non-cyberbullying risk participants (nCBY group; 70.1%; *n =* 450).

Variables	CBY		nCBY		*F*	*p*
	*M*	*SD*	*M*	*SD*		
Total scores of instruments						
SPAI-SF	5.51	2.75	3.57	2.71	67.45	***
SAS-SV	31.67	11.74	23.37	10.96	69.96	***
STDS	41.80	14.57	35.14	14.03	28.82	***
IAT	45.17	18.03	33.95	18.52	51.31	***
PIUQ-9	24.24	7.95	19.89	8.04	40.00	***
Online activities during weekdays (hours per day)						
Playing games	4.22	3.08	3.32	2.66	12.55	***
Using a cell phone (for any purpose)	6.03	3.08	5.03	2.67	15.32	***
Accessing the internet (e.g., social media, browsing)	5.88	3.22	5.06	2.95	9.26	**
Watching TV series	3.64	2.85	3.26	2.51	2.58	0.11
Online activities during weekends (hours per day)						
Playing games	3.99	3.39	3.50	3.18	2.87	0.09
Using a cell phone (for any purpose)	6.05	3.26	5.11	3.12	11.33	***
Accessing the internet (e.g., social media, browsing)	5.59	3.52	4.94	3.49	4.62	*
Watching TV series	3.67	3.11	3.09	2.89	4.89	*

*M,* mean; *SD,* standard deviation; *F,* one-way ANOVA; *p,* significance level. Weekdays: Monday–Friday; Weekends: Saturday–Sunday. *p < 0.05; **p < 0.01; ***p < 0.001.

**Table 10:** Profile of the SCOAC instrument and online activities and behaviors related to cyberbullying, aggressiveness, and reactive behaviors among adolescents classified as Cyberbullying risk participants (CBY group; 29.9%; *n =* 192) and non-Cyberbullying risk participants (nCBY group; 70.1%; *n =* 450).

Variables	CBY		nCBY		*F*	*p*
	*M*	*SD*	*M*	*SD*		
SCOAC						
Threat	6.61	5.72	0.60	1.21	208.0	***
Defamation / Exposure	8.65	5.77	0.96	1.65	330.0	***
Emotional Problems	5.48	4.90	0.42	1.09	201.0	***
Total scores	20.75	11.69	1.98	2.46	485.0	***
Florence Scale – Victimization						
Visual dimension	5.68	3.06	3.70	1.61	72.4	***
Falsification dimension	5.06	2.43	3.30	0.79	96.9	***
Exclusion dimension	7.16	3.44	4.04	1.89	139.6	***
Total score	17.90	6.74	11.03	3.12	182.5	***
Florence Scale – Aggression						
Visual dimension	4.43	2.57	3.24	0.82	39.4	***
Falsification dimension	4.42	2.41	3.26	1.06	41.2	***
Exclusion dimension	5.79	3.12	3.86	1.84	63.5	***
Total score	14.64	6.68	10.37	2.98	72.4	***
Q-CARP						
Aggressive Behaviors	12.13	4.62	9.72	4.35	38.0	***
Reactive Behaviors	24.18	9.69	19.13	8.88	38.5	***
Total score	36.31	13.19	28.84	12.28	44.9	***

*M,* mean; *SD,* standard deviation; *F,* one-way ANOVA; *p,* significance level. ***p < 0.001.

**Table 11:** Emotional and behavioral health profiles of adolescents categorized as cyberbullying risk participants (CBY group; 29.9%; n = 192) and non-cyberbullying risk participants (nCBY group; 70.1%; n = 450).

Variables	CBY		nCBY		*F*	*p*
	*M*	*SD*	*M*	*SD*		
Quality of Life (KIDSCREEN-10)						
Total score	30.34	8.01	30.94	8.58	0.74	0.39
DASS-21						
Depression	8.76	6.36	4.53	5.15	66.09	***
Anxiety	9.38	6.32	4.60	5.15	85.69	***
Stress	9.52	6.13	5.17	5.17	73.97	***
Emotion Regulation (DERS-18)						
Awareness	9.78	3.26	10.46	3.44	5.69	*
Clarity	8.10	3.46	6.35	3.33	35.31	***
Goals	8.55	3.87	6.28	3.45	49.25	***
Impulse	7.77	4.03	5.35	3.11	55.05	***
Nonacceptance	7.98	3.92	5.68	3.35	50.54	***
Strategies	7.67	3.87	5.46	3.16	48.84	***
Total score	49.85	15.48	39.59	12.97	64.98	***
Impulsivity (UPPS-P)						
Negative urgency	11.06	3.20	10.61	3.64	2.38	0.12
Lack of perseverance	8.13	2.68	7.75	2.97	2.58	0.10
Lack of premeditation	8.68	2.77	7.86	3.03	11.07	***
Sensation seeking	11.07	3.24	10.85	3.47	0.56	0.45
Positive urgency	10.58	3.09	10.14	3.75	2.37	0.12
Total score	49.52	6.60	47.22	7.94	14.39	***

*M,* mean; *SD,* standard deviation; *F,* one-way ANOVA; *p,* significance level. *p < 0.05; ***p < 0.001.

## 4 Discussion

In this study, we aimed to develop and gather psychometric evidence for the Scale of Cyberbullying and Online Aggressive Conduct (SCOAC), especially among Brazilian adolescents. Our main results indicate a strong internal structure (three factors/17 items) for the SCOAC, excellent confirmatory fit indices, high internal consistency, and invariance across genders. In the confirmation phase, using a more conservative estimation strategy also helped improve the stability of the final solution, especially after refining the set of items. Additionally, the ROC curve showed good accuracy in distinguishing adolescents with and without CYB. Overall, this evidence included criterion, convergent, and nomological validity, confirmed our hypotheses, and supported the SCOAC as a brief and reliable tool for identifying CYB risk among adolescents. We also recognize that discrepancies between some indicators observed during the exploratory phase and the best-fit obtained in the CFA should be understood in the context of the instrument’s refinement process. The EFA was initial and exploratory, conducted on a version still being refined. Conversely, the CFA was performed only after removing items with poor factorial performance and low centrality in the network analysis, and it was carried out on an independent subsample. Therefore, the CFA results reflect the instrument’s final, refined structure rather than a direct replication of the initial, rough exploratory solution.

The SCOAC has a three-factor structure, divided into behavioral patterns (Threat; Defamation/Exposure) and a central psychosocial impact (Emotional Problems). This dimensional framework aligns with the Lifestyle-Routine Activities Theory (L-RAT), which suggests that online routines that increase victims’ visibility or exposure and reduce parental protection or mediation can heighten the likelihood that motivated offenders will act (16, 38).

In this context, the power imbalance between victim and aggressor can lead to various emotional harms, which are partly represented by the items comprising the third dimension of the SCOAC (emotional problems). For example, multiple studies have shown an association between CYB and emotional problems (9), sleep disturbances (39), and difficulties in concentration and daily functioning (40), all of which are reflected in the items of this dimension. Regarding external validity, the criterion-related validity coefficients demonstrated strong correlations between the SCOAC total scores and the FCBVS scale. This is noteworthy because both scales assess core CYB behaviors in adolescents and share similar criteria, including intentionality, repetition/re-exposure, and power imbalance (41).

In terms of convergent validity, stronger correlations were observed, especially involving smartphone use measures (SAS-SV and SPAI-SF). These findings suggest that more extended smartphone use may be associated with a greater risk of CYB exposure. This is consistent with other research indicating that adolescents who access the internet through mobile devices are more likely to either perpetrate or experience virtual attacks (42). Regarding the psychosocial links with the SCOAC score (nomological validity), the highest correlation coefficients were observed for “anxiety” and “depression,” which demonstrated moderate strength.

These data support the link between CYB and emotional distress, especially traits like physiological and cognitive hyperactivation (43), which align with reports of persistent worry, hypervigilance (44), and rumination (45) that often occur with repeated online exposure. Among adolescents, these traits may be even more prominent due to increased psychosocial vulnerability during this developmental stage (46, 47). Some of these features were observed in this study, in which adolescents were divided into CYB (29.9%) and non-CYB (70.1%) groups based on the SCOAC cutoff point (≥ 8) established by ROC analysis. Adolescents in the CYB group showed significantly higher levels of anxiety and depression, greater impulsivity and emotional dysregulation, lower quality of life, and increased substance use involvement.

This study has some limitations. First, although the SCOAC was designed specifically for adolescents, its psychometric properties have not been evaluated in other age groups or among adolescent subgroups with varying internet use and digital exposure patterns. Second, we did not assess the temporal stability of the SCOAC (test-retest). Third, although the sample size (N = 642) is adequate, allocating 60% to EFA and 40% to CFA reduced the power of CFA and prevented invariance analysis across age groups. Fourth, the predictive validity of the SCOAC was evaluated indirectly through the LPA-derived classes. We chose this approach for two reasons: (i) during this study, the FCBVS was the only instrument adapted and validated in Brazil that measured CYB behaviors and could be compared with the SCOAC; (ii) because the FCBVS lacks a cutoff point, we needed to perform an LPA to identify cases and controls. For future research, we recommend that: (i) the SCOAC be translated, adapted, and validated in other languages; (ii) its accuracy, validity, including temporal stability, be tested; and (iii) its psychometric properties be examined in other populations, especially among young adults.

## Data Availability

The datasets presented in this study can be found in online repositories. The names of the repository/repositories and accession number(s) can be found below: https://osf.io/f3z5t/overview.

## References

[1] Hudon A Harvey E Nicolas S Dufour M Guérin-Thériault C Bérubé-Fortin J . Defining cyberpsychopathy: An integrative review. JMIR Ment Health. (2025), 10.2196/75167. doi: 10.2196/75167. PMID: 40154964

[2] Lee G Choi S . A comparative study on cyberbullying behaviors among Korean and American college students. Soc Sci. (2025) 14(5):257. doi: 10.3390/socsci14050257. PMID: 41725453

[3] Malta DC Souza JB Vasconcelos NM Mello FCM Buback JB Gomes CS . Cyberbullying among Brazilian schoolchildren: data from the National Student Health Survey, 2019. Ciênc Saúde Coletiva. (2024) 29(9):e19572023. doi: 10.1590/1413-81232024299.19572023. PMID: 39194122

[4] Kasturiratna KTAS Hartanto A Chen CHY Tong EMW Majeed NM . Umbrella review of meta-analyses on cyberbullying. Nat Hum Behav. (2024) 9:101–32. doi: 10.1038/s41562-024-02011-6. PMID: 39516404 PMC11774762

[5] Brazilian Internet Management Committee (BIMC) . TIC kids online Brasil. (2025). Available online at: https://cetic.br/media/docs/publicacoes/2/20250512154312/tic_kids_online_2024_livro_eletronico.pdf (Accessed November 2, 2025).

[6] Ramos RFS Scatena A Kim HS de Oliveira WA Andrade ALM . Brazilian Digital Warriors: Unraveling the Nexus of Adolescent Cyber Aggressors, Problematic Internet & Smartphone Use, Emotional Struggles, and Parental Mediation. Trends Psychol. (2023) 33(3):819–35. doi: 10.1007/s43076-023-00338-z. PMID: 41933263

[7] Xie Y Zhang M-M Wang C Cai J Wang Y Mu Y-F . The effect of bullying victimization on internet addiction: mediated by cyberbullying perpetration and moderated by social support. BMC Public Health. (2025) 25(1):2856. doi: 10.1186/s12889-025-23905-8. PMID: 40836288 PMC12366385

[8] Romualdo C de Oliveira WA Nucci LB Rodríguez Fernández JE da Silva LS Freires EM . Cyberbullying victimization predicts substance use and mental health problems in adolescents: data from a large-scale epidemiological investigation. Front Psychol. (2025) 16:1499352. doi: 10.3389/fpsyg.2025.1499352. PMID: 40357488 PMC12066598

[9] Zhang D Gong J Liu J Bullock A Sang B . Cyberbullying and depression: A systematic review. Aggress Violent Behav. (2025) 82:102052. doi: 10.1016/j.avb.2025.102052. PMID: 41936479

[10] Zhang W Huang S Lam L Evans R Zhu C . Cyberbullying definitions and measurements. Front Public Health. (2022) 10:1000504. doi: 10.3389/fpubh.2022.1000504. PMID: 36388377 PMC9642089

[11] Saleem S Khan NF Zafar S Raza N . Systematic literature reviews in cyberbullying. Technol Soc. (2022) 70:102055. doi: 10.1016/j.techsoc.2022.102055. PMID: 41936479

[12] Chun J Lee J Kim J Lee S . Systematic review of cyberbullying measurements. Comput Hum Behav. (2020) 113:106485. doi: 10.1016/j.chb.2020.106485. PMID: 41936479

[13] Lan M Law N Pan Q . Effectiveness of anti-cyberbullying programs. Comput Hum Behav. (2022) 130:107200. doi: 10.1016/j.chb.2022.107200. PMID: 41936479

[14] Polanin JR Espelage DL Grotpeter JK Ingram K Michaelson L Spinney E . A systematic review and meta-analysis of interventions to decrease cyberbullying perpetration and victimization. Prev Sci. (2022) 23(3):439–54. doi: 10.1007/s11121-021-01259-y. PMID: 34159506 PMC8218972

[15] Cohen LE Felson M . Routine activity approach. Am Sociol Rev. (1979) 44(4):588. doi: 10.2307/2094589

[16] Hindelang MJ Gottfredson MR Garofalo J . Victims of personal crime. (Cambridge: Ballinger) (1978).

[17] International Test Commission . Guidelines on Test Adaptation (2nd ed.). (2017). Available online at: https://www.intestcom.org/files/guideline_test_adaptation_2ed.pdf (Accessed November 2, 2025).

[18] American Educational Research Association APA NCME . Standards for educational and psychological testing. (Washington, DC: AERA) (2014).

[19] Conselho Federal de Psicologia (CFP) . Resolução CFP n° 31/2022. (2022). Available online at: https://normasbrasil.com.br/norma/resolucao-31-2022_473938.html (Accessed November 2, 2025).

[20] Cavalcanti JG Paiva TT Pimentel CE Pinto AVdL de Moura GB . Psychometric parameters of the Florence Scale Cyberbullying-Cybervictimization. Psico. (2019) 50(3):e31520. doi: 10.15448/1980-8623.2019.3.31520

[21] Brito AB Pinho L Brito MFSF Messias RB Brito KDP Rodrigues CAO . Propriedades psicométricas do Internet Addiction Test em estudantes de Montes Claros, Minas Gerais, Brasil. Cad Saúde Pública. (2021) 37(5):e00212619. doi: 10.1590/0102-311X00212619. PMID: 34008788

[22] Spritzer DT Machado WL Yates MB Astolfi VR Laskoski P Pessi C . Psychometric properties of the nine-item problematic internet use questionnaire in a Brazilian general population sample. Front Psychiatry. (2021) 12:660186. doi: 10.3389/fpsyt.2021.660186. PMID: 34054616 PMC8149803

[23] Andrade ALM Kim D-J Caricati VV Martins GDG Kirihara IK Barbugli BC . Validity and reliability of the Brazilian version of the Smartphone Addiction Scale-Short Version for university students and adult population. Estud Psicol (Campinas). (2020) 37:e190117. doi: 10.1590/1982-0275202037e190117. PMID: 41880456

[24] Andrade ALM Scatena A de Oliveira Pinheiro B de Oliveira WA Lopes FM De Micheli D . Psychometric properties of the Smartphone Addiction Inventory (SPAI-BR) in Brazilian adolescents. Int J Ment Health Addict. (2021) 20(5):2690–705. doi: 10.1007/s11469-021-00542-x. PMID: 41933263

[25] Spritzer DT Andrade ALM Xavier AZ da Silva GT Kim HS Kaliszewska-Czeremska K . The self-perception of text message dependence scale (STDS): A Brazilian-Portuguese validation and expansion of its psychometric properties. Curr Psychol. (2022) 42(21):17670–81. doi: 10.1007/s12144-022-02957-8. PMID: 35291222 PMC8914152

[26] Patias ND Machado WDL Bandeira DR Dell'Aglio DD . DASS-21 for Brazilian adolescents. Psico-USF. (2016) 21(3):459–69. doi: 10.1590/1413-82712016210302. PMID: 41880456

[27] Machado YC de Paula JJ Malloy-Diniz LF Miranda DM Romano-Silva MA . Psychometric properties of the Brazilian Portuguese version of the UPPS-P impulsive behavior scale for children and adolescents. J Pediatr. (2023) 99(6):588–96. doi: 10.1016/j.jped.2023.04.008. PMID: 37263340 PMC10594007

[28] Cancian ACM Souza LAS Silva VHPe Machado WdL Oliveira MdS . Psychometric properties of the Brazilian version of the Difficulties in Emotion Regulation Scale (DERS). Trends Psychiatry Psychother. (2019) 41(1):18–26. doi: 10.1590/2237-6089-2017-0128. PMID: 30234889

[29] Borsa JC Bandeira DR . Comportamentos agressivos e reativos. Psico-USF. (2014) 19(2):287–96. doi: 10.1590/1413-82712014019002015. PMID: 41880456

[30] Braccialli LMP Almeida VS Sankako AN Silva MZ Braccialli AC Carvalho SMR . Translation and validation of the Brazilian version of the Cerebral Palsy Quality of Life Questionnaire for Children – child report. J Pediatr. (2016) 92(2):143–8. doi: 10.1016/j.jped.2015.05.005. PMID: 26699433

[31] Pereira BAX Schram PFC Azevedo RCS . Escala CRAFFT/CESARE. Ciênc Saúde Coletiva. (2016) 21(1):91–9. doi: 10.1590/1413-81232015211.05192015. PMID: 26816167

[32] Cheung GW Rensvold RB . Goodness-of-fit indexes. Struct Equ Model. (2002) 9(2):233–55. doi: 10.1207/S15328007SEM0902_5. PMID: 38329648

[33] Cho E . Making reliability reliable. Organ Res Methods. (2016) 19(4):651–82. doi: 10.1177/1094428116656239. PMID: 41930703

[34] Dunn TJ Baguley T Brunsden V . From alpha to omega. Br J Psychol. (2014) 105(3):399–412. doi: 10.1111/bjop.12046. PMID: 24844115

[35] Schober P Boer C Schwarte LA . Correlation coefficients: Appropriate use and interpretation. Anesth Analg. (2018) 126(5):1763–8. doi: 10.1213/ane.0000000000002864. PMID: 29481436

[36] Smits N Smit F Cuijpers P De Graaf R . Optimal cutoff scores using decision theory. Int J Methods Psychiatr Res. (2007) 16(4):219–29. doi: 10.1002/mpr.230. PMID: 18188835 PMC6878391

[37] Khoury JM de Freitas AAC Roque MAV Albuquerque MR das Neves MCL Garcia FD . Assessment of the accuracy of a new tool for the screening of smartphone addiction. PLoS One. (2017) 12(5):e0176924. doi: 10.1371/journal.pone.0176924. PMID: 28520798 PMC5435144

[38] Aizenkot D . Predictability of routine activity theory. J Interpers Violence. (2021) 37(13-14):NP11857–NP11882. doi: 10.1177/0886260521997433. PMID: 33636999

[39] Wang W Xie M Liu Z Chen H Wu X Lin D . Linking daily victimization to daily affect among adolescents: The mediating role of sleep quality and disturbance. J Youth Adolesc. (2024) 54(2):354–67. doi: 10.1007/s10964-024-02076-6. PMID: 39251472

[40] Solas-Martínez JL Rusillo-Magdaleno A Garrote-Jurado R Ruiz-Ariza A . Cyberbullying and study time. Educ Sci. (2025) 15(5):563. doi: 10.3390/educsci15050563. PMID: 41725453

[41] Palladino BE Nocentini A Menesini E . Florence cyberbullying scales. Cyberpsychol Behav Soc Netw. (2015) 18(2):112–9. doi: 10.1089/cyber.2014.0366. PMID: 25599108

[42] Schulz PJ Boldi MO van Ackere A . Adolescent cyberbullying and cyber victimization: Longitudinal study before and during COVID-19. J Med Internet Res. (2025) 27:e70508. doi: 10.2196/70508. PMID: 40132197 PMC11979530

[43] Suanrueang P Peltzer K Lkhamsuren Z Yap LK . Psychosocial factors and distress. Sci Rep. (2023) 13(1). doi: 10.1038/s41598-023-39452-4. PMID: 37537238 PMC10400538

[44] McLoughlin LT Simcock G Schwenn P Beaudequin D Driver C Kannis-Dymand L . Cyberbullying, metacognition, and quality of life: preliminary findings from the Longitudinal Adolescent Brain Study (LABS). Discov Psychol. (2022) 2(1). doi: 10.1007/s44202-021-00013-3. PMID: 41933263

[45] Jiang H Jin Y Yang Q . Bullying and anger rumination. Psychol Res Behav Manag. (2025) 18:877–86. doi: 10.2147/PRBM.S507510. PMID: 40226436 PMC11992003

[46] Silva RVdS Dias Moura HS Betetti PNA Santos FLd Fortuna CM . Virtual violence as a contributing factor to the mental health of young people: A scoping review. Can J Nurs Res. (2025) 57(4):480–96. doi: 10.1177/08445621251364528. PMID: 40801920

[47] García-Fernández CM Moreno-Moya M Ortega-Ruiz R Romera EM . Adolescent involvement in cybergossip: Influence on social adjustment, bullying and cyberbullying. Span J Psychol. (2022) 25:e6. doi: 10.1017/sjp.2022.3. PMID: 35105416

