# Supplementary material for: Development and validation of a scale of cyberbullying and online aggressive conduct in Brazilian adolescents
Source: Front Psychiatry. 2026 May 5;17:1759871. doi: 10.3389/fpsyt.2026.1759871 (PMC13183630; doi:10.3389/fpsyt.2026.1759871)
Supplement: Supplementary file 1 [file Table1.docx]

**Appendix 1.** Scale of Cyberbullying and Online Aggressive Conduct - SCOAC (English Version)

**Instructions:** Thinking about the last 2–3 months, how many times did each of the following situations happen to you?

| **Scale of Cyberbullying and Online Aggressive Conduct - SCOAC** | | | | | |
| --- | --- | --- | --- | --- | --- |
| **Threat** | **Never (0)** | **One time (1)** | **Two times (2)** | **Three times (3)** | **Four or more times (4)** |
| **1.** Someone made fun of me, insulted me, or called me names online. | 0 | 1 | 2 | 3 | 4 |
| **2.** I felt threatened or unsafe because of something that happened online. | 0 | 1 | 2 | 3 | 4 |
| **3.** Someone threatened to hurt me physically on the internet. | 0 | 1 | 2 | 3 | 4 |
| **4.** Someone threatened to spread lies about me online. | 0 | 1 | 2 | 3 | 4 |
| **5.** Someone threatened to exclude me from something online. | 0 | 1 | 2 | 3 | 4 |
| **6.** Someone said they would get back at me or take revenge on me online. | 0 | 1 | 2 | 3 | 4 |
| **Defamation / Exposure** |  |  |  |  |  |
| **7.** Someone shared a photo or video of me without my permission. | 0 | 1 | 2 | 3 | 4 |
| **8.** Someone gave me a mean or nasty nickname online. | 0 | 1 | 2 | 3 | 4 |
| **9.** Someone made a meme or edited a picture about me. | 0 | 1 | 2 | 3 | 4 |
| **10.** Someone posted a screenshot of my private conversation without asking me. | 0 | 1 | 2 | 3 | 4 |
| **11.** Someone shared my personal information (such as my address or phone number) without my permission. | 0 | 1 | 2 | 3 | 4 |
| **12.** Someone told a secret or shared something private I said in messages or on social media. | 0 | 1 | 2 | 3 | 4 |
| **Emotional Problems** |  |  |  |  |  |
| **13.** Being attacked online is messing with my mental and emotional health. | 0 | 1 | 2 | 3 | 4 |
| **14.** It has been hard to focus since I started getting mean messages or comments online. | 0 | 1 | 2 | 3 | 4 |
| **15.** It has been hard to sleep since I started getting mean messages or comments online. | 0 | 1 | 2 | 3 | 4 |
| **16.** It has been hard to do everyday things during the day since I started getting mean messages or comments online. | 0 | 1 | 2 | 3 | 4 |
| **17.** I have noticed changes in my mood or behavior since I started getting mean messages or comments online. | 0 | 1 | 2 | 3 | 4 |

**Correction instructions:** All items are scored in the same direction, without the need for inversion. The higher the score, the more severe the symptoms. We suggest a cutoff of ≥ 8 for classifying cyberbullying.

**Statement of use, translation, and validation:** The English version of the scale is freely available for clinical, scientific, and educational purposes. We authorize its reproduction, translation, adaptation, and validation in other countries and languages, without prior permission, provided the source of this manuscript is cited.

Interested researchers may also perform back-translations and, if desired, forward them to the corresponding author, Dr. André Luiz Monezi Andrade (last author), for quality assessment against the original English version.

**Appendix 2.** Escala de Comportamento Online Agressivo e Cyberbullying -*ECOAC* *(Brazilian version).*

**Instruções:** Pensando nos últimos 2 a 3 meses, quantas vezes cada uma destas situações aconteceu com você?

| **Escala de Comportamento Online Agressivo e Cyberbullying (ECOAC)** | | | | | | |
| --- | --- | --- | --- | --- | --- | --- |
| **Ameaça** | **Nunca (0)** | **Uma vez**  **(1)** | **Duas vezes (2)** | **Três vezes (3)** | **Quatro ou mais vezes (4)** |  |
| **1.** Fui alvo de piadas, ofensas ou xingamentos online | 0 | 1 | 2 | 3 | 4 |  |
| **2.** Fui ameaçado ou me senti inseguro por causa das minhas interações online | 0 | 1 | 2 | 3 | 4 |  |
| **3.** Recebi algum tipo de ameaça física na internet | 0 | 1 | 2 | 3 | 4 |  |
| **4.** Recebi algum tipo de ameaça de difamação (disseminar informações falsas) na internet | 0 | 1 | 2 | 3 | 4 |  |
| **5.** Recebi algum tipo de ameaça de ser excluído de algo na internet | 0 | 1 | 2 | 3 | 4 |  |
| **6.** Recebi algum tipo de ameaça de retaliação na internet (vingança) | 0 | 1 | 2 | 3 | 4 |  |
| **Difamação/exposição** |  |  |  |  |  |  |
| **7.** Compartilharam alguma foto ou vídeo meu sem o meu consentimento. | 0 | 1 | 2 | 3 | 4 |  |
| **8.** Criaram algum apelido ruim ou maldoso contra mim na internet. | 0 | 1 | 2 | 3 | 4 |  |
| **9.** Criaram algum meme ou montagem a meu respeito. | 0 | 1 | 2 | 3 | 4 |  |
| **10.** Compartilharam alguma captura de tela (print) de uma conversa minha com alguém sem meu consentimento. | 0 | 1 | 2 | 3 | 4 |  |
| **11.** Divulgaram alguma informação pessoal minha (endereço, telefone, etc.) sem o meu consentimento. | 0 | 1 | 2 | 3 | 4 |  |
| **12.** Revelaram algum segredo ou comentário em aplicativos de mensagens ou redes sociais. | 0 | 1 | 2 | 3 | 4 |  |
| **Problemas emocionais** |  |  |  |  |  |  |
| **13.** Ser alvo de ataques virtuais está afetando minha saúde mental e emocional. | 0 | 1 | 2 | 3 | 4 |  |
| **14.** Tenho dificuldades para me concentrar desde que comecei a receber mensagens ou comentários ofensivos na internet. | 0 | 1 | 2 | 3 | 4 |  |
| **15.** Tenho dificuldades para dormir desde que comecei a receber mensagens ou comentários ofensivos na internet. | 0 | 1 | 2 | 3 | 4 |  |
| **16.** Tenho dificuldades para realizar as atividades diárias desde que comecei a receber mensagens ou comentários ofensivos na internet. | 0 | 1 | 2 | 3 | 4 |  |
| **17.** Notei mudanças em meu comportamento ou humor desde que comecei a receber mensagens ou comentários ofensivos na internet. | 0 | 1 | 2 | 3 | 4 |  |

**Instruções de correção:** Todos os itens são pontuados na mesma direção, sem necessidade de inversão. Quanto maior a pontuação, maior a gravidade dos sintomas. Sugerimos um ponto de corte ≥ 8 para a classificação de cyberbullying.

**Statement of use and validation:** The Brazilian version of the scale is freely available for clinical, scientific, and educational purposes. We authorize its reproduction, adaptation, and validation in Portuguese-speaking countries, without prior permission, provided the source of this manuscript is cited.
